# Response surface method for polyhydroxybutyrate (PHB) bioplastic accumulation in *Bacillus drentensis* BP17 using pineapple peel

**DOI:** 10.1371/journal.pone.0230443

**Published:** 2020-03-19

**Authors:** Watsana Penkhrue, Dieter Jendrossek, Chartchai Khanongnuch, Wasu Pathom-aree, Tomoyasu Aizawa, Rachel L. Behrens, S. Lumyong

**Affiliations:** 1 Research Center of Excellence in Microbial Diversity and Sustainable Utilization, Department of Biology, Faculty of Science, Chiang Mai University, Chiang Mai, Thailand; 2 School of Preclinic, Institute of Science, Suranaree University of Technology, Nakhon Ratchasima, Thailand; 3 Institute of Microbiology, University of Stuttgart, Stuttgart, Germany; 4 Division of Biotechnology, Faculty of Agro-Industry, Chiang Mai University, Chiang Mai, Thailand; 5 Faculty of Advanced Life Science, Hokkaido University, Sapporo, Japan; 6 Global Institution for Collaborative Research and Education, Hokkaido University, Sapporo, Japan; 7 Polymer Facility Technical Director, UCSB, MRL, Santa Barbara, CA, United States of America; 8 Academy of Science, The Royal Society of Thailand, Bangkok, Thailand; Universitat Jaume I de Castelló, SPAIN

## Abstract

Polyhydroxybutyrate (PHB) is a biodegradable biopolymer which is useful for various applications including packing, medical and coating materials. An endospore-forming bacterium (strain BP17) was isolated from composted soil and evaluated for PHB production. Strain BP17, taxonomically identified as *Bacillus drentensis*, showed enhanced PHB accumulation and was selected for further studies. To achieve maximum PHB production, the culture conditions for *B*. *drentensis* BP17 were optimized through response surface methodology (RSM) employing central composite rotatable design (CCRD). The final optimum fermentation conditions included: pineapple peel solution, 11.5% (v/v); tryptic soy broth (TSB), 60 g/L; pH, 6.0; inoculum size, 10% (v/v) and temperature, 28°C for 36 h. This optimization yielded 5.55 g/L of PHB compared to the non-optimized condition (0.17 g/L). PHB accumulated by *B*. *drentensis* BP17 had a polydispersity value of 1.59 and an average molecular weight of 1.15x10^5^ Da. Thermal analyses revealed that PHB existed as a thermally stable semi-crystalline polymer, exhibiting a thermal degradation temperature of 228°C, a melting temperature of 172°C and an apparent melting enthalpy of fusion of 83.69 J/g. It is evident that *B*. *drentensis* strain BP17 is a promising bacterium candidate for PHB production using agricultural waste, such as pineapple peel as a low-cost alternative carbon source for PHB production.

## Introduction

Bioplastics are a popular and environmentally-friendly alternative to conventionally synthesized plastics, as they are made from renewable biostock and can be composted. Currently, polyhydroxyalkanoates (PHAs), polylactic acid (PLA), and polybutylene adipate-co-terephthalate (PBAT) are well-known biodegradable polyesters and are commercially available in the market [1]. Microorganisms accumulated PHAs in the form of granules as an energy and carbon storage source. PHAs accumulation, normally occur when nitrogen sources are limited but carbon sources are abundant in the culture medium [2–5]. PHAs are biodegradable thermoplastics that can be produced from sugars, agricultural wastes, glycerol and vegetable oil [6–8] and can be degraded by microorganisms in aerobic conditions. The resulting products from biodegradation are mainly water and carbon dioxide which makes [9, 10]. PHAs favorable as an alternative bioplastic instead of petroleum-derived plastics. PHAs bioplastics can be versatilely utilized in medicine and agriculture owing to their biocompatibility and biodegradability characteristics [11, 12].

Polyhydroxybutyrate (PHB), a currently well-studied type of PHAs, was first discovered by Lemoigne in 1925 [13]. PHB is a biodegradable plastic with properties similar to synthetic plastics and has a molecular weight (Mw) of 1×10^4^–3×10^6^ Da, exhibiting a polydispersity of around 2 [14]. The glass transition temperature, densities of crystalline and amorphous PHB are 180°C, 1.26 and 1.18 g (cm^3^)^-1^, respectively [15]. Although the tensile strength and Young’s modulus of PHAs are close to polypropylene (PP), the extension to break (5%) for PHB is however lower than that of PP (400%) [16, 17]. The production costs of PHAs depend on many factors including microorganisms, substrates used, media, cultivation, recovery, and purification processes. Gram-positive bacteria, *Bacillus* was reported as the first PHB-producing bacteria [13]. Among them, *Bacillus megaterium* S29 and *Bacillus* sp. IPCB-403 accumulated over 70% PHB content per cell dry weight in optimal conditions [18, 19]. *Bacillus aryabhattai* T34-N4 utilized waste starch for direct production of PHB [20]. Moreover, *Bacillus cereus* is reported to yield up to 48% of PHB content when using starch as a carbon source [21]. Genus *Bacillus* is suggested to be one of the microorganisms that are suitable for PHB production since it obtains high PHB yields and needs few fermentation factors [22]. The response surface method (RSM) has been applied to enhance the PHAs yield in several *Bacillus* species such as *Bacillus mycoides* DFC1 [23], *B*. *megaterium* SRKP-3 [24], *Bacillus safensis* EBT1[25] and several strains of *Bacillus subtilis* [26, 27]. Therefore, RSM is a suitable tool for improving the yield of PHAs.

In addition to the type of microorganism used, the use of inexpensive substrates has become increasingly important because the substrate or carbon source is the major cost factor in PHAs production [28]. For instance, Dalsasso et al. used vinasse and sugarcane molasses as substrate for PHB production [29, 30]. Additionally, a new *Methylobacterium* isolate produced 0.55 g/L PHB using methanol as a sole carbon source under two-stage fermentation [31]. Currently, petroleum-derived plastics have lower total production costs than PHAs [32], but many studies using agricultural and industrial waste materials have shown through statistical optimization that using waste material could lower PHAs production cost [33–35].

Another example of agricultural waste is pineapple waste. Pineapple wastes, which are obtained from the canned pineapple industry includes residual peel, pulp, and skin, consist of mainly sugar, cellulose, hemicelluloses and other carbohydrates, which provide the necessary nutrients for PHB production. These wastes are in abundance in many countries such as Thailand, who is a major exporter of canned fruits, Thailand produces 0.62 million tons of industrial fruit wastes per year [36].

In this study, we aim to isolate and identify PHAs-producing bacteria, optimize medium components by utilizing waste from pineapples, and cultivation conditions using RSM to improve PHAs production as well as PHAs characterization.

## Materials and methods

### Sample collection and bacterial isolation

Forty soil samples were collected for PHAs-producing bacteria isolation from various sites in Chiang Mai (lat. 18°46'57.1'' N and long. 98°57'14.7'' E), Kamphaeng Phet (lat. 16°26'9.4'' N and long. 99°18'16.2'' E), Phayao (lat. 19°18'3.6'' N and long. 100°7'41.6'' E) and Udon Thani (lat. 17°14'55.6'' N and long. 102°42'33.0'' E) province, Thailand. All samples were collected between April-July, 2014. Sampling sites included: termite mould (TM), leaf composted (LC) and agricultural (AG) soil. Soil samples were taken from 30 cm depth. One gram of each soil sample was processed by serial dilution spread plate technique on mineral salt medium (MSM) agar with 1% (w/v) cellulose [37].

#### Screening of isolates for PHAs producers

For PHAs staining with Nile red fluorescence dye, all 83 isolates were cultured on MSM medium [37] containing 1% (w/v) glucose and 10 μg/mL Nile red (Sigma-Aldrich, MO, USA) then incubated at 30°C for 3 days [38]. Under UV light, bright orange fluorescent colonies were selected. In the secondary screening, 6 isolates that exhibited intense fluorescence were confirmed for strong PHAs accumulation using gas chromatography and PHAs granules accumulation using fluorescence microscopy.

#### Fluorescence microscopy

PHAs granules and PolyP granules were stained with 1 μg/mL Nile red and 60 μg/mL of 4, 6-diamidino-2-phenylindole (DAPI), respectively. Fluorescence microscopy (Leica Microsystems, Germany) was used for PHAs and PolyP granules observation follow by Jendrossek and co-workers [39]. PHAs granules and polyP became visible as red and yellow fluorescent globular structures, respectively.

#### Determination of the amount of PHAs

Six PHAs-accumulating bacteria were cultured in MSM medium containing 1% (w/v) glucose. The culture flasks were incubated at 30°C on rotary 150 rpm shaker for 7 days. The bacterial cells were collected for PHAs content analysis using gas chromatography (GC) according to Lageveen et al. [40]. For this assay, the PHB polymer (Sigma-Aldrich, Munich, Germany) and benzoic acid methyl ester (Sigma-Aldrich, Munich, Germany) were used as an external standard and internal standard, respectively.

### Morphological, cultural and physiological characterization

Bacterial strain BP17 cell morphology was determined using a light microscope (Olympus CX31RBSF, Tokyo, Japan) and a JEOL scanning electron microscope (SEM) (Model JSM-5910LV, JOEL Ltd., Tokyo, Japan). The biochemical characterization of strain BP17 was done using API kit 20E, API ZYM and API Coryne medium (Biomérieux Co. USA). Gram staining and motility test were carried out according to Vos et al. [41]. Growth at different temperatures was determined in tryptic soy agar (TSA) at 10, 20, 25, 30, 37, 45, 50 and 60°C for 24 h. BP17 cells were tested for salt tolerance on 0, 1.0, 2.0, 3.0, 4.0, 5.0, 6.0, 7.0, 8.0, 9.0 and 10.0% (w/v) NaCl-containing TSB agar at 30°C for 24 h. The pH ranges for growth (pH 4.0, 5.0, 6.0, 7.0, 8.0, 9.0, 10.0, 11.0 and 12.0) were determined using TSA plates. The growth at various temperature, NaCl concentrations and pH were examined for growth at 24 h.

### Molecular identification using 16S rRNA sequence and phylogenetic analysis

The 16S rRNA gene was amplified using universal primers (27F 5'-AGAGTTTGATCMTGGCTCAG-3' and 1492R 5'-TACGGYTACCTTGTTACGACTT-3') and ligated in pGEM-T Easy Vector (Promega, Madison, WI, USA), transformed to *Escherichia coli* JM109 cells and finally the DNA sequence was determined at 1^st^ BASE Laboratory (Singapore). The 16S rRNA gene was analyzed and phylogenetic trees were reconstructed using the MEGA software version 10.0 as described by Watsana et al. [42].

### Optimization of experimental design and statistical analysis

PHAs bioplastic was produced in 250 mL Erlenmeyer flasks containing 50 mL culture media and inoculated with 10% (v/v) inoculum. Then, the flasks were incubated at 30°C for 72 h and centrifuged at 6,000x*g* for 15 minutes. The biomass pellets were freeze dried. The amount of the polymer in the bacterial cell was quantified using gas chromatography (Agilent Technologies, MA, USA).

#### Juice extraction process

All fruit peels (dragon fruit, apple, pineapple, mango, sugarcane, and banana) were cut and dried at 60°C for 3 days then ground to obtain a fine powder. The powder was extracted using the hot water method with a ratio of 5:80 (dry fruit peel powder: distilled water; w/v) as described by Kumar et al.[43]. Extraction was carried out at 95°C for 15 min. Afterwards, the supernatant was collected by centrifugation (6,000 rpm, 10 min) and filtrated through a 0.45 μm filter paper. The final volume of the juice was adjusted to 100 mL.

#### Effect of medium on PHAs production

For the selection of the best medium for PHAs production, various production medium (nutrient broth; NB, casein yeast magnesium broth; CYM, tryptic soy broth; TSB, glucose broth; GB, Luria Bertani broth; LB, peptone water; PW, potato dextrose broth; PDB, Sabouraud dextrose broth; SDB, yeast extract-peptone dextrose broth; YPD, yeast and mold broth; YM, minimal salt medium; MSM and peptone broth; PEP) were tested to examine the optimal PHAs production from *Bacillus drentensis* BP17. All media were supplemented with 1% (w/v) glucose, and PHAs yield was determined.

#### Effect of carbon source on PHAs production

One percentage (w/v) of each carbon source (glucose, fructose, and sucrose) or 1% (w/v) total reducing sugar of extracted fruit juice (dragon fruit, apple, pineapple, mango, sugarcane and banana) were added into 50 mL of the TSB medium for *Bacillus drentensis* BP17 cultivation. Bacterial cells were collected and freeze-dried before PHAs yield assay.

#### Central composite rotatable design (CCRD)

CCRD was applied to obtain the optimal medium for further enhancement of PHAs production. The best carbon source (extract pineapple peel juice; EPPJ) and the best medium (TSB medium, Merck, Germany) were chosen for further optimization. Two factors, which included three replicates at five center points, were used to fit a second-order response surface resulting in 13 experiments (**Table 1**). All experiments were conducted in biological triplicate in Erlenmeyer flasks containing 50 mL medium at 150 rpm for 3 days. The PHAs contents were analyzed using gas chromatography and the observed values were analyzed and fitted to the second-order model equation as follows in Eq (1) [44]:

**Table 1:** Experimental design matrix and responses for optimization of PHAs content using CCRD.

Run	TSB medium, *x*_*i*_ (g/L)	EPPJ, *x*_*j*_ (%)	PHAs yield (g/L)	
			Experimental	Predicted
1	18.00	5.00	0.36	0.57
2	100.00	5.00	1.56	2.06
3	18.00	20.00	1.74	1.31
4	100.00	20.00	0.06	-0.077
5	1.02	12.50	0.40	0.57
6	116.98	12.50	0.90	0.66
7	59.00	1.89	2.30	1.81
8	59.00	23.11	0.40	0.82
9	59.00	12.50	4.20	4.18
10	59.00	12.50	4.00	4.18
11	59.00	12.50	3.88	4.18
12	59.00	12.50	4.40	4.18
13	59.00	12.50	4.40	4.18

TSB medium was purchased from Becton, Dickinson and Company, MD, USA.

EPPJ = Extracted pineapple juice.

$$Y=\beta_{0}+\sum \beta_{i}x_{i}+\sum \beta_{ii}x_{i}{}^{2}+\sum \beta_{ij}x_{i}x_{j}$$ (1)

Where *Y* is the variable response (PHAs content (% w/w)); *x*_*i*_ and *x*_*j*_ are the independent factors of the experiment (TSB medium and EPPJ); *β*_0_ is the intercept term; *β*_i_, *β*_i_ and *β*_ij_ are the linear, squared and interaction coefficients, respectively. The analysis of variance (ANOVA) was performed and the model terms was deemed significant when “Prob > *F*” values were less than 0.05. The non-significant of lack-of-fit *F*-value is considered a good model. The model was validated with respect to the PHAs content under the predicted medium components from the significant model under the conditions for 7 days.

### Effect of initial pH on PHAs production

The optimized medium (50 mL) was adjusted to an initial pH between 5.0 to 10.0. For fermentation, the optimized medium containing EPPJ (11.5% (v/v)) as carbon source and TSB medium (60 g/L) were added in 250 mL Erlenmeyer flask and sterilized. Seed inoculum (10%, v/v) of *B*. *drentensis* BP17 was transferred in each flask and incubated at 30°C on a shaker, 150 rpm for 72 h. The cells were centrifuged, lyophilized and dried pellets were used for PHAs assays.

#### Effect of inoculum size for PHAs production

Fifty mL of optimized medium containing TSB medium (60 g/L) and EPPJ (11.5% (v/v)) in 250 mL Erlenmeyer flask was adjusted with an initial pH of 6.0. A total of 4, 6, 8 and 10, 20% (v/v) seed inoculum was transferred into each flask. The optimal incubation temperature was maintained at 30°C on 150 rpm shaker. After 36 h, the culture was centrifuged and the cells were collected for PHAs yield assay.

### Effect of temperature for PHAs production

The test flask 250 mL contained TSB medium (60 g/L) and EPPJ (11.5% (v/v)) and the 10% (v/v) of inoculum was transferred into the optimal medium. Then, the flasks were incubated at a different temperature: 25, 28, 30, 35 and 37°C for 36 h. The cultures were centrifuged; the dried bacterial cells were assayed for PHAs yield.

### PHAs biosynthesis under optimal condition

Sterile NB medium (8 g/L) was used as an inoculum preparation medium for *B*. *drentensis* BP17. The seed culture was incubated on a shaker at 150 rpm, 30°C for 18 h. The 10% (v/v) of inoculum was transferred into the optimized TSB broth with EPPJ (TSB medium, 60 g/L and EPPJ, 11.5% (v/v)), and the flasks were incubated at 28°C for 36 h. The PHAs content was analyzed using gas chromatography (GC) [40]. Biomass dry weight was prepared from the standard calibration of the absorbance of 600 nm and known biomass dry weight using Thermo scientific Genesys 20 spectrophotometer (Thermo Fisher Scientific, USA) [45]. The initial and residual concentrations of glucose were quantified in centrifuged culture supernatant samples by high performance liquid chromatography (HPLC) apparatus (Agilent Technologies 1260 InfinityII, Frankfurt, Germany), equipped with APS-2-HYPERSIL column (250 mm x 46 mm length, Thermofisher, MA, USA) and 2414 RI detector. Mobile phase [acetonitrile-water (75:25)] was used at 1.0 mL/min and the sample was injected in duplicate [46]. Total nitrogen quantification in the culture broth was performed using an Analytikjena multi N/C 2100S according to Guzman Lagunes and Winterburn [47].

### PHAs polymer extraction

PHAs were extracted from *B*. *drentensis* BP17 cultivated in the optimized medium (pH 7.0) at 30°C with 150 rpm shaking for 72 h. PHAs granules were extracted according to Teeka et al. [48] using 5% (v/v) sodium hypochlorite (Wako Chemical, Japan) extraction. The purified PHAs were washed twice with methanol followed by distilled water and dried in a vacuum machine at 40°C and its purity was checked using GC analysis.

### Characterization of PHAs

Isolate BP17 was cultured in both TSB medium containing EPPJ and NB medium containing glucose. The cell biomass was harvested and extracted for PHAs powder. The polymer obtained using EPPJ and glucose as substrates were characterized and the data obtained were compared with standard PHB which was analyzed under the same conditions.

#### The Fourier transform infrared spectroscopy

FT-IR spectrum was measured using Nicolet 6700 FT-IR spectrometer (Thermo Scientific, United States). The samples were scanned between a wavenumber range 4,000–400 cm^-1^ with a total of 64 scans per sample at a resolution of 4 cm^-1^[49].

#### X-ray diffraction

XRD measurement of PHB was performed at room temperature on RIGAKU SmartLab X-ray Diffractometer employing nickel-filtered Cu Kα radiation (λ = 1.5406 E; 40 kV, 30 mA) in the 2*Ɵ* range of 2–50° at 25°C, using a scan speed of 10°/min. The degrees of polymers crystallization were estimated from the XRD spectra.

#### The nuclear magnetic resonance

Samples of produced PHB and standard PHB (Sigma, Germany) were dissolved in deuterated chloroform (CDCl_3_) and NMR spectra were recorded using a JNM-ECA600 NMR spectrometer (JEOL, Japan) at 20°C. The signals of tetramethylsilane (TMS) were used as the standards for a chemical shift of ^1^H spectra.

#### Number and weight average molecular mass

Gel permeation chromatograms (GPC), analyzed at the University of California, Santa Barbara (UCSB), were acquired on a Water Acquity Advanced Polymer Chromatography (APC) system equipped with an Acquity RI detector and three 4.6 mm × 75 mm Water Acquity APC XT-extended temperature columns (pore diameters: 45 Å, 200 Å and 450 Å). The mobile phase consisted of chloroform with 0.25% (v/v) triethylamine (TEA) flowing at 0.5 mL/min. The column temperature was fixed at 35°C.

#### Thermogravimetric analysis

TGA was used to determine the decomposition temperature (T_d_) of PHB using a Seiko EXSTAR 6000 TG/DTA 6300 thermal analyzer in an aluminum pan. PHB powder was heated from 40-600°C with a heating rate of 20°C/min under nitrogen atmosphere.

#### Differential scanning calorimetry

DSC (METTLER DSC823e thermal analyzer) was used to analyze the melting temperature (T_m_), thermal crystallization temperature (T_c_) and glass transition temperature (T_g_) for all polymer samples. The temperature ranges of DSC varied from -50°C to 250°C (1^st^ cycle: heating at room temperature to 250°C; 2^nd^ cycle: cooling from 250°C to -50°C and 3^rd^ cycle; heating from -50°C to 250°C) at a heating rate of 10°C/min and a cooling rate of 5.0°C/min. The STARe Evolution software was used for data analysis.

#### The melting enthalpy

The polymer crystallinity (X_p_) was calculated from ΔH_m_ based on the melting enthalpy (∆H_m_°) of 100% crystallinity PHB as reported in Dai et al. [50]. The melting enthalpy of 100% crystalline PHB is assuming 142 J/g as cited in the literature [51].

### Statistical analysis

All experiments (effects of media, carbon sources, pH, inoculum, and temperature) were performed as biological and technical triplicates and data were statistically analyzed using one-way ANOVA and Ducan’s multiple comparison test with the aid of software SPSS Version 17.0 (International Business Machines Corporation (IBM), New York, US). The *p*-values less than 0.05 were used as the cut-off for statistical significance. The Design Expert Software version 7.0.0 (Stat-Ease Corporation, Minneapolis, USA) was used to design the experiments, ANOVA statistical analysis, process optimization, the regression and graphical analysis of the CCRD experiment data.

## Results and discussion

Using agricultural wastes is a promising choice as a carbon source for microbial PHAs production. Microorganisms have the capability for both accumulating PHAs and producing cellulase which is advantage for producing PHAs in cellulose-containing medium. The isolation of new microbial species from the abundant cellulosic materials habitats is a one of the most efficient methods for isolating cellulose-degrading microbes with PHAs accumulating ability. Currently, no known reports have described the isolation of PHAs-producing bacteria with cellulase activity. Therefore, this study aimed to isolate and screen PHAs-producing bacteria from cellulose-containing soil and optimized medium for PHAs production.

A total 83 bacterial isolates were obtained from 40 soil samples in Chiang Mai (CM), Kamphaeng Phet (KP), Phayao (PY) and Udon Thani (UT) province of 3 soil types (Termite mould (TM), Leaf composted (LC), and Agricultural (AG) soil). The primary screening, identified 6 isolates exhibited a high intensity of fluorescence under UV light after staining with Nile red (S1 Fig). 16S rRNA analysis of these 6 strains showed that they belonged to genus *Shinella*, *Mycoplana*, and *Bacillus*. More than 70 bacterial and archaea genera have been reported as PHAs-producing microbes [52–54]. *Bacillus* species are renowned sources of PHAs polymer that were mostly found to produce short-chain PHAs and PHAs content between about 2 and 50% [55]. Among these, strain BP17 exhibited the maximum intensity of fluorescence under UV light after staining with Nile red, PHAs granules, and PHAs content of 19.9% (S1 Table). Therefore, the strain BP17 was selected for further optimization of PHAs production.

**Fig 1:**
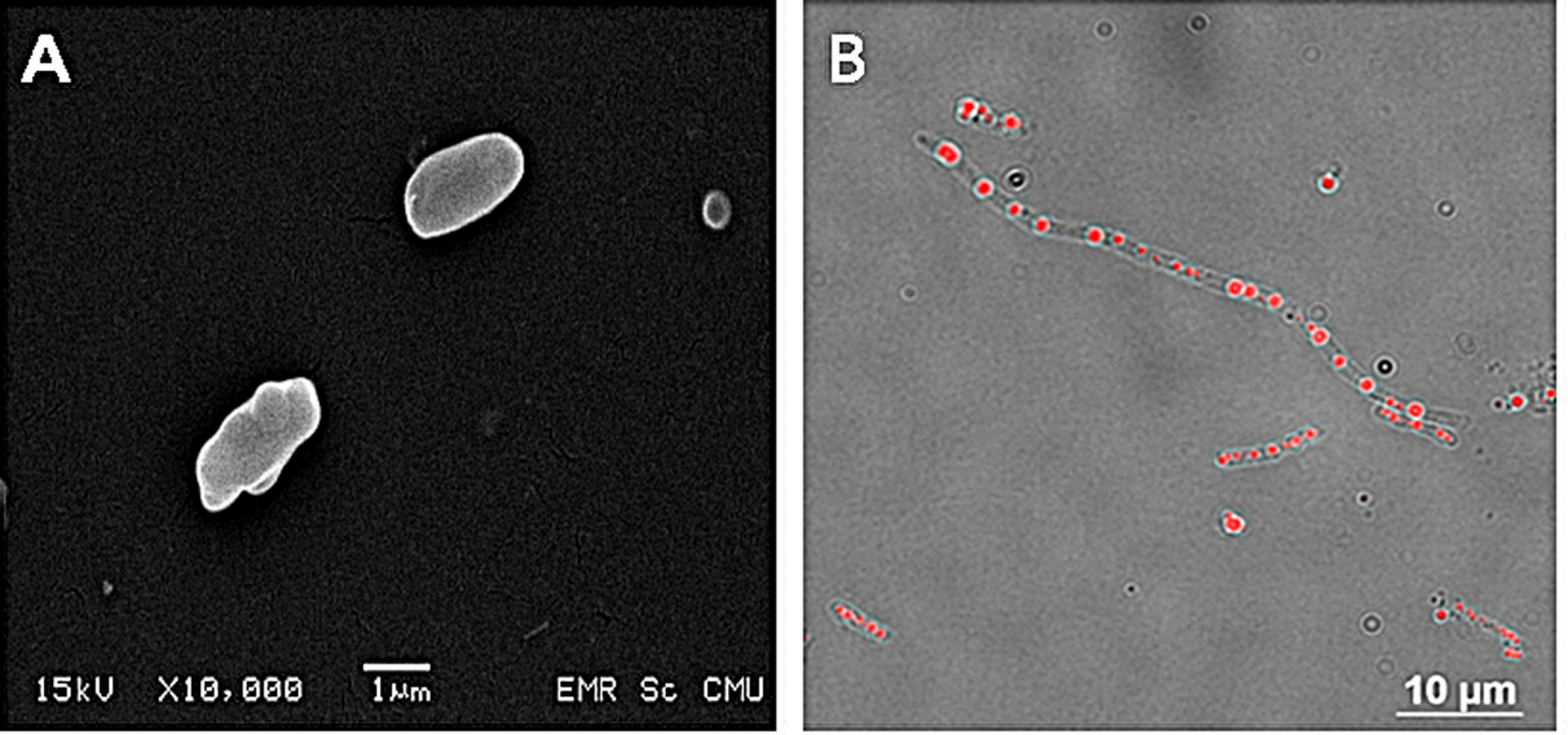
*Bacillus drentensis* BP17 cells exhibiting PHAs inclusion. (A) Scanning electron microscope (SEM) when grown on nutrient agar (NA) plate at 30°C for 24 h and (B) Nile red staining under fluorescence microscope when cultivated in Nutrient broth containing 1% (w/v) glucose at 30°C, 150 rpm for 24 h.

Isolate BP17 exhibited morphological, physiological and biochemical characteristics of the genus *Bacillus*. Cells of the isolated bacterial strain (BP17) were Gram-positive, aerobic, non-motile, and rod-shaped (1.5–10.0 x 0.5–1.0 μm) forming endospores (**Fig 1A**). The presence of PHAs granules was confirmed by Nile red staining (**Fig 1B**). Colonies on nutrient agar (NA) were slightly convex with regular margins with pale yellow colonies. The optimum temperature for growth was 37°C and the maximum growth temperature expressed at 45°C. Strain BP17 tolerated abroad pH spectrum (pH 5.0 to 12.0), but most growth occurred between pH 6.0 and 8.0. The strain could not grow on 3% (w/v) NaCl. The o-nitrophenyl-*β*-D-galactopyranoside (ONPG) hydrolysis was positive, and the nitrate reduction was variable. The Voges-Proskauer reaction and reactions for arginine dihydrolase, citrate utilization, gelatin hydrolysis, hydrogen sulfide production, indole production, lysine decarboxylase, ornithine decarboxylase, tryptophan deaminase, and urease were negative. Strain BP17 utilized D-glucose, D-sucrose, D-xylose, D-melibiose, D-lactose, D-maltose, D-mannitol, L-arabinose and amygdalin as a source of carbon and energy (S2 Table).

**Fig 2:**
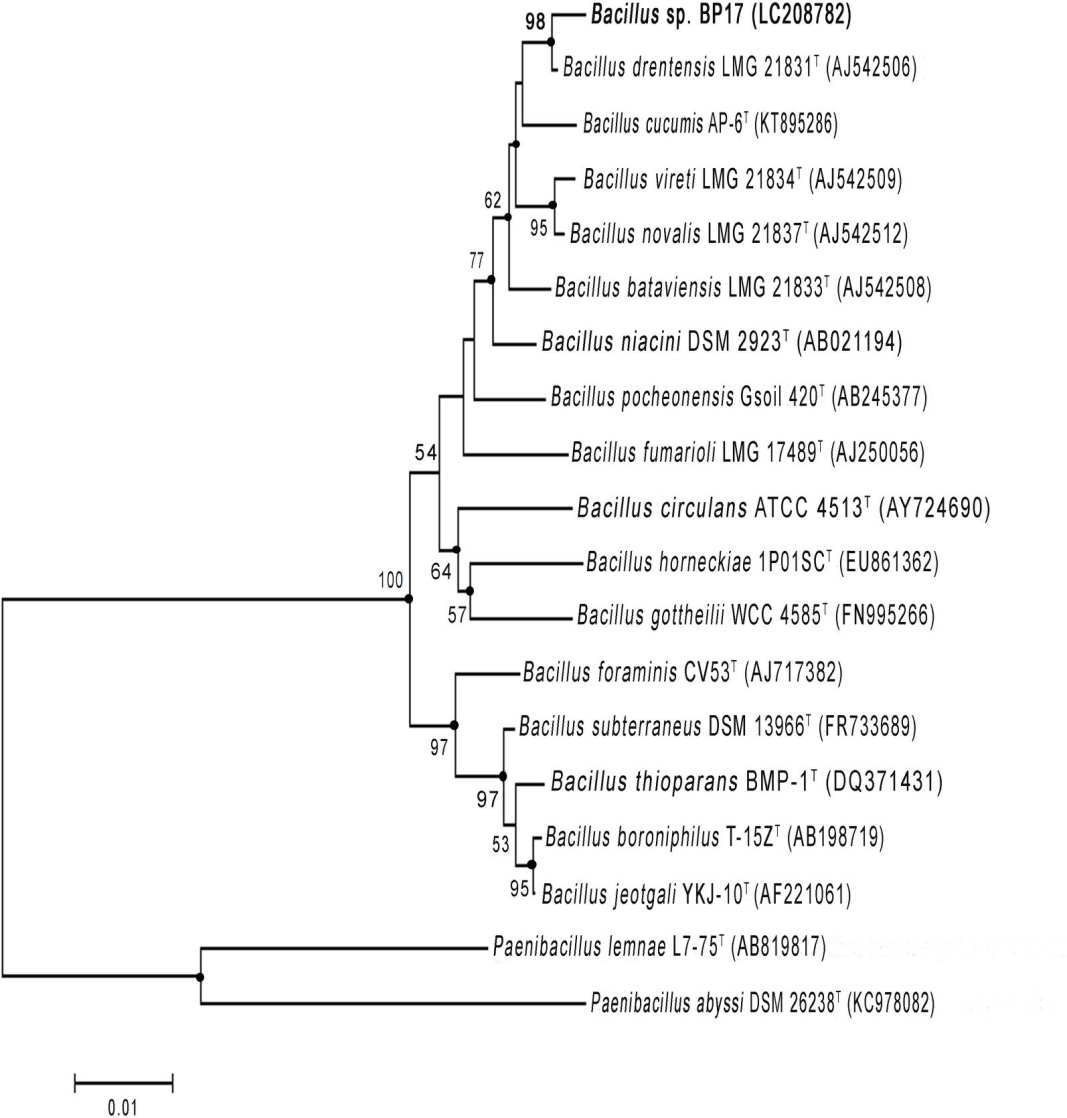
Neighbor-joining tree based on 16S rRNA gene sequences showing phylogenetic relationship between strain BP17 and closely related taxa of the genus *Bacillus*. *Paenibacillus lemnae* L7-75^T^ and *Paenibacillus abyssi* DSM 26238^T^ were used as outgroup. Only bootstrap values above 50% (percentages of 1000 replications) are shown. Nodes marked with filled circle indicate branches were also recovered in the reconstructed trees using the maximum-likelihood and maximum-parsimony method; Bar 0.01 nucleotide substitutions per site.

The 16S rRNA gene sequence of strain BP17 (1,470 nt) has been deposited in the DDBJ database under the accession number LC208782 and is referred to as *Bacillus* sp. BP17. The result of EzTaxon-e analysis revealed that strain BP17 should be classified into the genus *Bacillus* with the highest sequence similarities to *B*. *drentensis* LMG 21831^T^ (99.2%), *B*. *niacini* IFO 15566^T^ (98.3%), *B*. *bataviensis* LMG 21833^T^ (98.0%), *B*. *cucumis* AP-6^T^ (97.9%), *B*. *vireti* LMG 21834^T^ (97.8%) and *B*. *novalis* NBRC 102450^T^ (97.8%), which was supported by a 77% bootstrap value in the neighbor-joining phylogenetic tree (**Fig 2**). The tree topology in this region was also supported by the maximum-likelihood and the maximum-parsimony algorithm. The strain BP17 was clustered with most known strains of *Bacillus* sp. In conclusion, the genotypic and phenotypic data of strain BP17 suggest that strain BP17 is a member of the species *Bacillus drentensis* LMG 21831^T^ [56].

Species of *Bacillus* have been widely studied for PHAs production due to its absence of a lipopolysaccharides (LPS) endotoxin since the discovery of PHB in *B*. *megaterium* [14, 52]. However, *B*. *drentensis* had not been previously reported as a species that could produce PHAs.

### Optimization of experimental design and statistical analysis

The media composition and conditions are minor variations, which can be tuned to optimize the yields and the metabolic profile of microorganisms [57]. A statistical method of experimental design is necessary to facilitate the selection of the optimal culture conditions. Among the various media studied, *B*. *drentensis* BP17 produced a maximum PHAs yield in trypticase soy broth (TSB) medium (3.53 g/L), followed by Luria Bertani broth (LB) medium (2.72 g/L) and yeast extract-peptone dextrose broth (YPD) medium (2.55 g/L). Strains of the genus *Bacillus* showed less growth and PHB production in nutrient-limited conditions when compared with the nutrient medium but the percentage of sporulation was high [23]. Previous publications reported that sporulation is involved with the SpoOA gene during nutrient limitation [58] and the degradation of the PHB polymer [59]. The present study also supported the interference of PHB production by sporulation in *B*. *drentensis* BP17 when the bacteria were cultured in mineral salt medium (nutrient limitation).

Among the tested carbon sources, extracted pineapple peel juice (EPPJ) (3.44 g/L) enhanced maximum PHAs yield, followed by sucrose (2.77 g/L), and glucose (2.42 g/L) performed best with respect to PHB accumulation (**Table 2**). Considering the sugar-based materials of agricultural wastes that could be possibly applied for PHAs production by microorganisms, pineapple waste is lucrative a substrate for PHAs production. Previously, pineapple wastes were used as substrates for the production of antioxidants, ethanol, methane, citric acid and PHAs [60–64]. Consequently, the production cost of PHAs would be reduced by inexpensive substrates using like agricultural wastes especially, pineapple peel. This study used the environmentally-friendly pretreatment method for preparing pineapple peel waste. Many previous publications abundantly used multiple supplements and chemical pretreatment substrates [29, 60, 65–67]. To the best of the author's knowledge, there is only one report on PHAs production by microorganisms using chemical pretreated pineapple peel as substrates [60]. In a recently publication, it was reported that kenaf biomass could be used for PHB production by *Ralstonia eutropha* ATCC 17699. However, it was necessary to use various pretreatments of the raw material [66]. However, there are no reports of the synthesis of PHAs using pineapple peel without chemical pretreatment. Thus, TSB medium and EPPJ were selected as the medium and carbon source, respectively, for the design of response surface methodology.

**Table 2:** Effect of various media and carbon sources on PHAs production by *Bacillus drentensis* BP17 after 72 h at 30°C under shaking (150 rpm).

Nutrient source	PHAs yield (g/L)
**Medium:**	
Nutrient broth (NB)	0.55±0.002^h^
Casein yeast magnesium broth (CYM)	2.35±0.025^d^
Trypticase soy broth (TSB)	3.53±0.004^a^
Glucose broth (GB)	0.13±0.001^i^
Luria Bertani broth (LB)	2.72±0.001^b^
Peptone water (PW)	0.06±0.002^j^
Potato dextrose broth (PDB)	1.32±0.000^f^
Sabouraud dextrose broth (SDB)	2.22±0.009^e^
Yeast extract-peptone dextrose broth (YPD)	2.55±0.001^c^
Yeast and mold broth (YM)	0.90±0.001^g^
Minimal salt medium (MSM)	0.17±0.018^i^
Peptone broth (PEP)	0.10±0.003^i^
**Carbon source:**	
Glucose	2.42±0.042^b^
Fructose	2.04±0.305^c^
Sucrose	2.77±0.205^b^
Dragon fruit peel	0.52±0.018^d^
Apple peel	1.14±0.057^d^
Pineapple peel	3.44±0.082^a^
Mango peel	2.04±0.071^d^
Sugarcane peel	0.56±0.028^d^
Banana peel	0.36±0.020^d^

The mean and standard error per treatment were calculated from technical and biological triplicates. Lowercase letters represent significant differences at the 5% probability level. Significant differences were analyzed by the Duncan test, using SPSS 17.0.

The medium and conditions can be optimized by RSM to enhance the PHAs yield [23, 24, 68]. The CCRD experiments were designed to find the optimal level of TSB medium and EPPJ for the maximum yield of PHAs production of strain BP17. The quadratic regression equation for evaluation of the highest PHAs production is presented in terms of the actual value as follows in Eq (2):

$$PHAsyield(g/L)=-4.67+0.15x_{i}+0.73x_{j}–2.34x_{i}x_{j}-1.06x_{i}{}^{2}-0.02x_{j}{}^{2}$$ (2)

**Fig 3:**
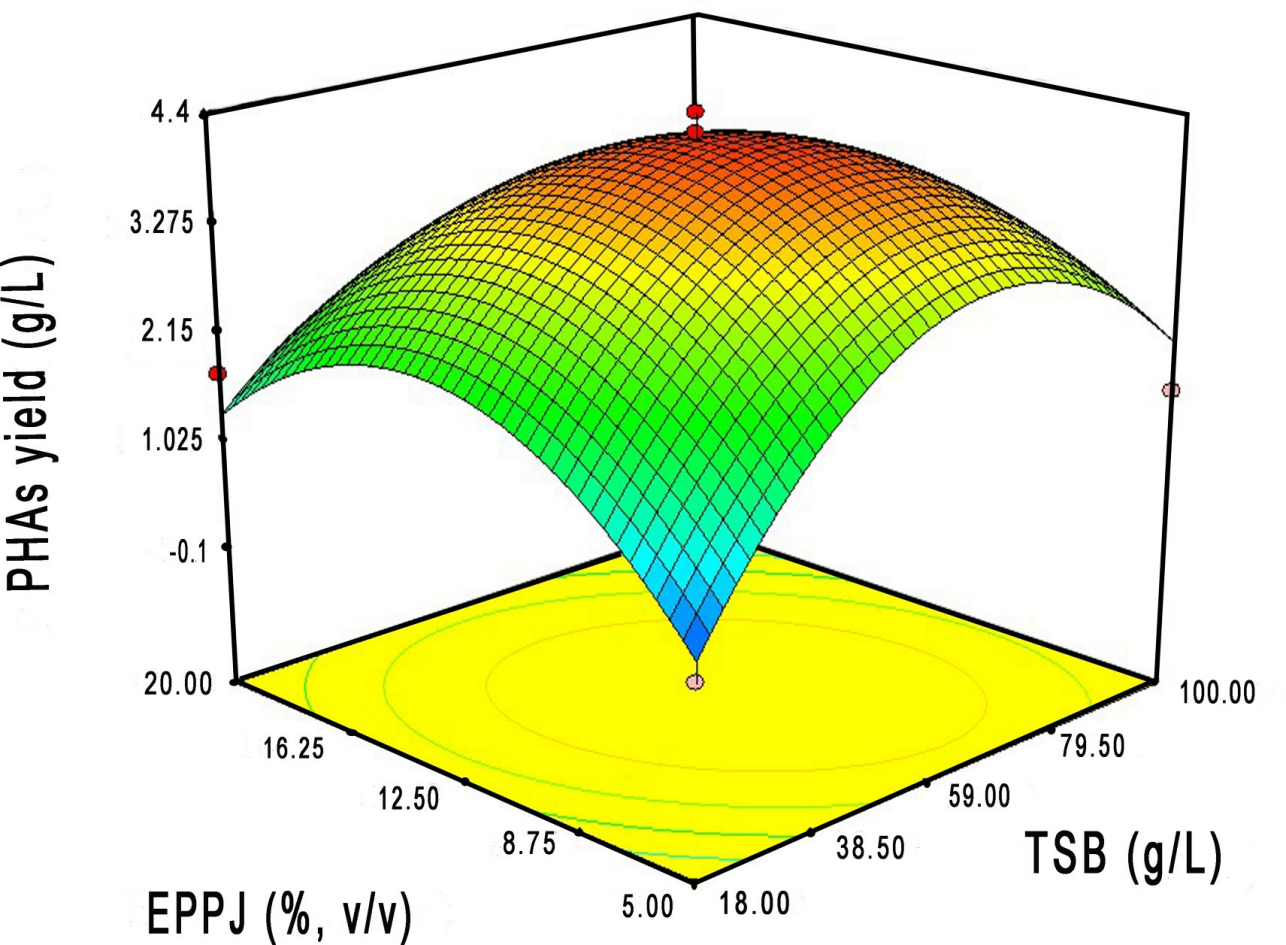
Response surface plot and contour showing the effect of TSB medium and extracted pineapple peel juice (EPPJ) on the PHAs production by *Bacillus drentensis* BP17.

Where *x*_*i*_ is the quantity of the TSB medium (g/L) and *x*_*j*_ is the quantity of the EPPJ (v/v), PHAs content fitted significantly into the quadratic model at *p* < 0.0001 with a reliability rating of *R*^*2*^ = 0.9663 (**Table 3**). Lack of fit was found to be insignificant. The convex shape of the 3D response surface plot indicated the optimum level of NB medium and glucose concentration and the maximum yield of the PHAs (**Fig 3**). According to the model analysis, the optimum ratio between TSB medium and EPPJ was 60 g/L and 11.5% (v/v), respectively. Verification experiments were conducted to validate the quadratic model under the predicted optimal conditions for PHAs production. The actual experimental data indicated that the PHAs yield was 3.9 g/L on cell dry weight (CDW), which was 4.1 g/L for PHAs production on 72 h of incubation time in agreement with the predicted model. Considering the time course of PHAs production, the yield of 4.0 g/L was obtained from the optimized medium after 36 h, decreasing production time by 50%. The RSM model had a significant effect on the improve PHAs production by *B*. *drentensis* BP17.

**Table 3:** ANOVA analysis of TSB medium and extracted pineapple peel juice (EPPJ) effects on PHAs production after fitting with response surface quadratic model.

Source	Coefficient	Sum of squares	df	Mean square	*F*-value	*p*-value Prob>*F*
Model		35.28	5	7.06	40.18	< 0.0001
*xi*-TSB medium	0.028	0.006	1	0.006	0.037	0.8535
*xj*-EPPJ	-0.35	0.98	1	0.98	5.61	0.0497
*xixj*	-0.72	2.07	1	2.07	11.81	0.0109
*xi*^*2*^	-1.78	22.05	1	22.05	125.59	< 0.0001
*xj*^*2*^	-1.43	14.24	1	14.24	81.07	< 0.0001
Residual		1.23	7	0.17		
*Lack of Fit*		1.01	3	0.34	6.13	0.0561
*Pure Error*		0.22	4	0.055		
Cor Total		36.51	12			

Standard deviation = 0.42; mean = 2.20; coefficient of variance (C.V. %) = 19.05; PRESS = 7.52; R-squared = 0.9663;

Adj R-squared = 0.9423; Pred R-squared = 0.7939; Adeq Precision = 14.941

The effect of initial pH was investigated for the optimized medium (S2A Fig), and it was observed that the difference in the production of PHAs was statistically significant in the pH range between 5 to 10. The maximum PHB yield (3.7 g/L) occurred at 36 h with the initial pH of the medium being pH 6.0, followed by 3.3 and 3.2 g/L of PHAs yield when *B*. *drentensis* BP17 was grown at pH 6.5 and 7.0, respectively. This result is similar to the study of the PHB production from *Comamonas* sp. [68] and *B*. *megaterium* A1 [69]. Acidic pH has no to effect on PHB degradation which is known to depolymerize under alkaline pH conditions [70] and sporulation [71]. Thus, the medium (pH 6.0) is suitable for PHAs production. Moreover, the effect of initial inoculum density on PHAs production by *B*. *drentensis* BP17 was studied by testing five different inoculum sizes: 4, 6, 8, 10 and 20% (v/v) in the optimized medium. This study suggests that inoculum size (2% (v/v)) of the medium was responsible for less growth and PHAs production. The yield of PHAs increased significantly from 3.1 to 4.1 g/L as the size of inoculum increased from 4 to 10% (v/v) of the medium (S2B Fig). PHAs accumulated in *B*. *drentensis* BP17 were assessed at various temperatures to estimate PHAs yield (S2C Fig). The strain produced the highest yield of PHAs when grown on the optimized medium with maximum accumulation at 28°C (5.0 g/L). At temperatures above 40°C, the bacteria could not grow.

### PHAs biosynthesis under optimal condition

**Fig 4:**
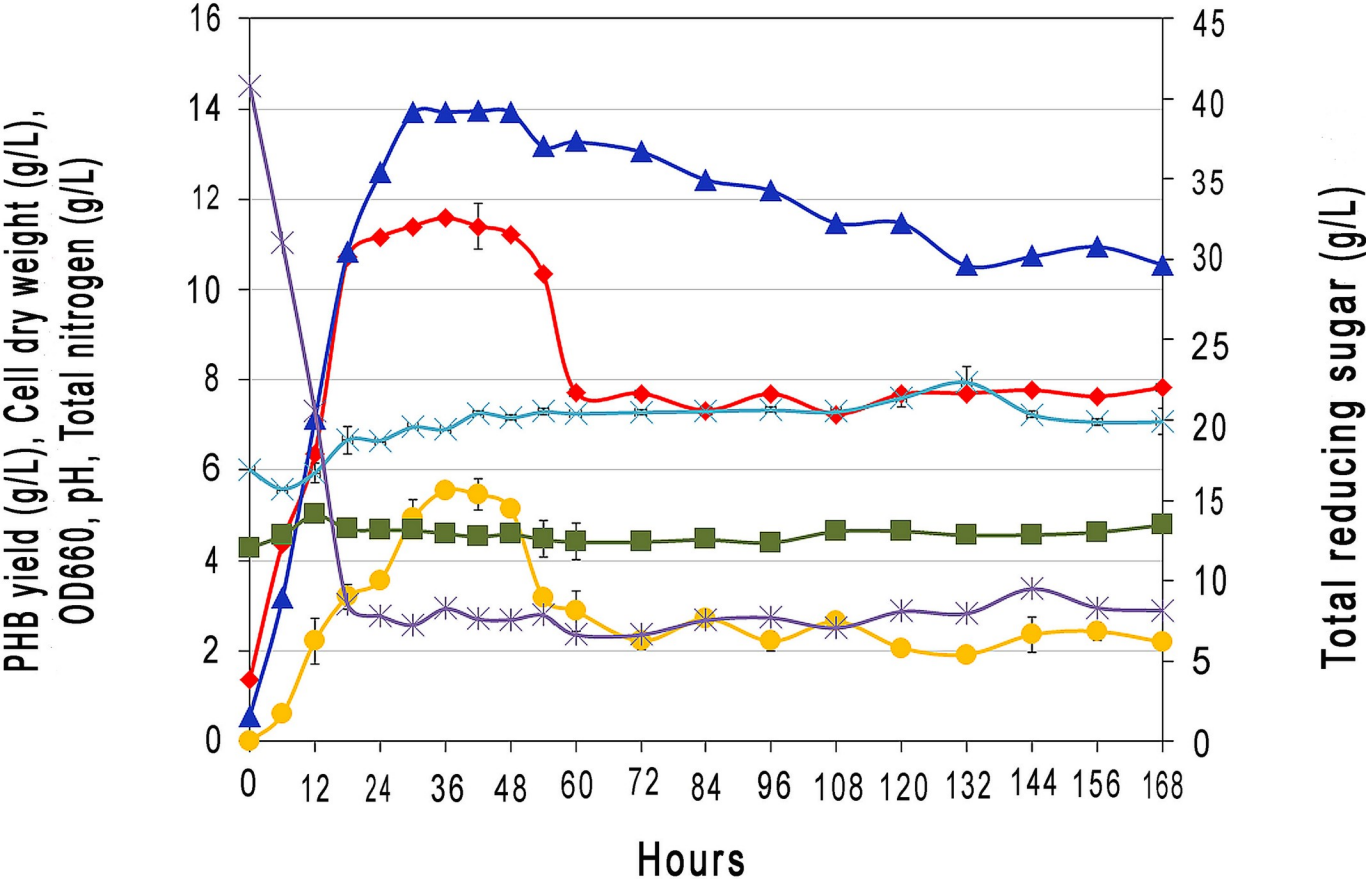
Time course of cell growth and PHAs accumulation of *Bacillus drentensis* BP17 grown in submerged culture of optimized medium with EPPJ as carbon source and TSB medium: (◆) Cell dry weight (g/L); (▲) OD600; (■) Total nitrogen (g/L); (✳) Total reducing sugar (g/L); (×) pH and (●) PHAs yield (g/L).

PHAs production of strain BP17 in optimized TSB broth containing EPPJ medium was investigated in batch cultivation. **Fig 4** shows that the log phase lasted approximately 24 h, and the culture reached the stationary phase after 48 h. The optimized medium contained 4.29 g/L of total nitrogen and 40.8 g/L of total reducing sugar. It appeared that the highest PHAs yield (5.6 g/L) was achieved after 36 h. To date, the highest PHAs yield of 3.32 g/L was reported from batch culture of *B*. *mycoides* DFC1 using glucose-peptone medium [23] and the PHB yield was determined to be 0.81 g/L when rice bran was used as a substrate using *B*. *subtilis* G-3 [27]. Interestingly, this study obtained PHAs yield higher than *B*. *megaterium* VB89, the yield of PHAs was 0.672 g/L [72]. The pH of the culture medium increased slightly from 6.0 to 7.0 in 7 days. The residual sugar in the culture medium of 8.2 g/L indicated that *B*. *drentensis* BP17 can utilize sugar for its growth and PHAs production. When compared with Li et al., only 3.3 g/L of PHB were obtained from multiple supplements including oxidative enzyme, mediators, surfactants and silicon nanoparticles which had been added to alkaline pretreatment liquor for *Cupriavidus necator* culture [65].

### Characterization of PHAs

After the polymer from the bacterial cell in EPPJ was extracted using sodium hypochlorite solution, the obtained polymer powder appeared to be white. When compared with previous publications using cassava waste, the cultured cells appeared light brown due to carotenoid pigment from cassava waste hydrolysate [67]. The polymers obtained from EPPJ and glucose were characterized using FTIR, XRD, NMR, GPC, GSC, and TGA analysis. The results of FTIR, XRD, and NMR analysis are very similar to standard PHB and previous reports including PHB extracted from *Bacillus cereus* CFR06 [73], *B*. *megaterium* VB89 [72] and *B*. *mycoides* DFC1 [23]. FTIR spectrum obtained for all polymer shows the similar main peaks at 1,720.6 cm^-1^ and 1,277–978.7 cm^-1^ which correspond to different functional groups in PHB. S3 Fig shows the peak at 1,277–978.7 cm^-1^ corresponding to C-C, C-O, and C-H group. The peak at 1,720 cm^-1^ which corresponds to C = O stretch of the ester group. Absorption bands occurring at 2,976.1 and 2,933.4 cm^-1^ indicate as aliphatic–CH_3_ and–CH_2_ groups, respectively.

The XRD pattern of a polymer obtained from EPPJ, glucose and standard PHB was determined using XRD analysis and is represented in S4 Fig. Both polymers exhibited a characteristic peak at 2*θ* values of 13, 17, 20, 21, 22 and 25. These values are similar to 2*θ* values of standard PHB (13.55, 17.05, 20.04, 21.61, 22.55 and 25.59). All these peaks possess narrow FWHM, indicating that these are the highly crystalline PHB. The XRD pattern of the polymers obtained from EPPJ and glucose matched well with the standard PHB and showed high crystallinity.

**Fig 5:**
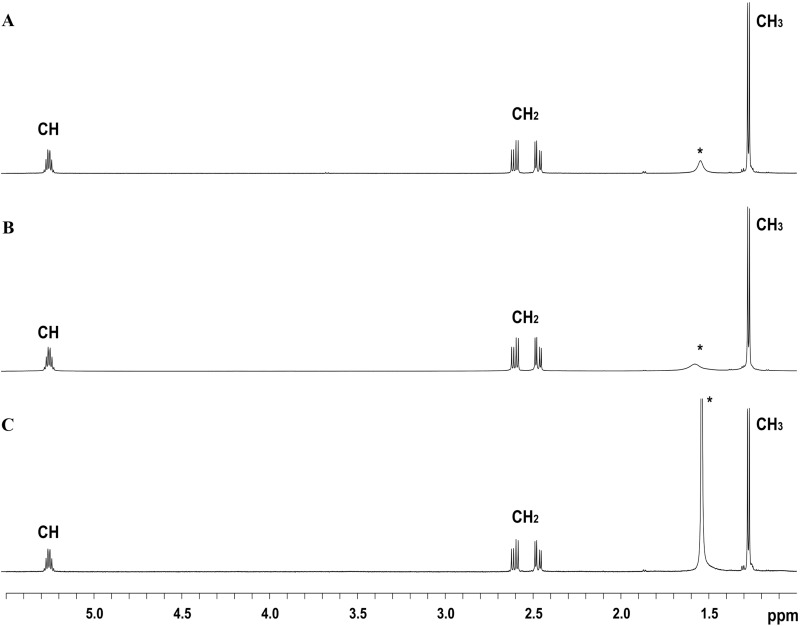
600 MHz ^1^H nuclear magnetic resonance spectrum of the PHAs obtained from EPPJ (A), glucose (B) and standard PHB (C). The main resonances occurred at 1.27, 2.45–2.65 and 5.25 ppm homopolymer. The peak at 1.56 ppm (*) is water.

The ^1^H NMR scans of all polymers obtained from *B*. *drentensis* BP17 using EPPJ and glucose as a substrate are represented in **Fig 5**. The monomeric composition of the PHAs was determined by ^1^H NMR spectrum. NMR spectrographs of all polymers exhibited a pattern of signal virtually identical to the spectrum of the PHB homopolymer with main resonances at 1.27, 2.45–2.65 and 5.25 ppm of methyl (CH_3_), diastereotopic methylene (CH_2_) and methine (CH) end groups, respectively. The NMR spectrum of the BP17 polymer showed similar patterns with those of PHB published elsewhere [48, 74, 75]. According to Jan and co-workers, the peak at 1.56 ppm and 5.25 ppm are the characteristic peak of water and PHB, respectively [76]. Therefore, these findings validate that the polyester accumulated by *B*. *drentensis* BP17 in this study is indeed PHB.

GPC analysis of all-polymer was performed to determine the molar distribution. In TSB medium containing EPPJ, the PHB polymer extracted from *B*. *drentensis* BP17 has an average molecular weight (Mw), number average molecular weight (Mn), and polydispersity index (PDI) defined as Mw/Mn, 1.15x10^5^ Da, 7.26x10^4^ Da and 1.59, respectively. The PHAs obtained from NB containing glucose had a relatively high average of Mw (3.46x10^5^ Da), Mn (2.02 x10^5^ Da) and low PDI (1.71) compared to standard PHB (Mw = 2.64 x10^5^ Da, Mn = 8.24 x10^4^ Da, PDI = 3.21). According to the previous report, Mw and Mn value of PHAs showed a wide range from 5.0x10^4^ Da to 1.0×10^7^ Da and PDI could be in a range between 1.1 and 6.0 [52, 77–79]. PHB obtained from EPPJ in the present study has a low Mw and low polydispersity index of 1.59 compared to PHB obtained from glucose, standard PHB and other polymers reports [52, 72]. Many studies have shown that bioplastics have good biodegradability, making them suitable for biomedical applications such as an embedding material drug [72, 80]. So, PHB obtained from this study is likely suitable for packaging and tissue engineering applications because of its low polydispersity index.

**Fig 6:**
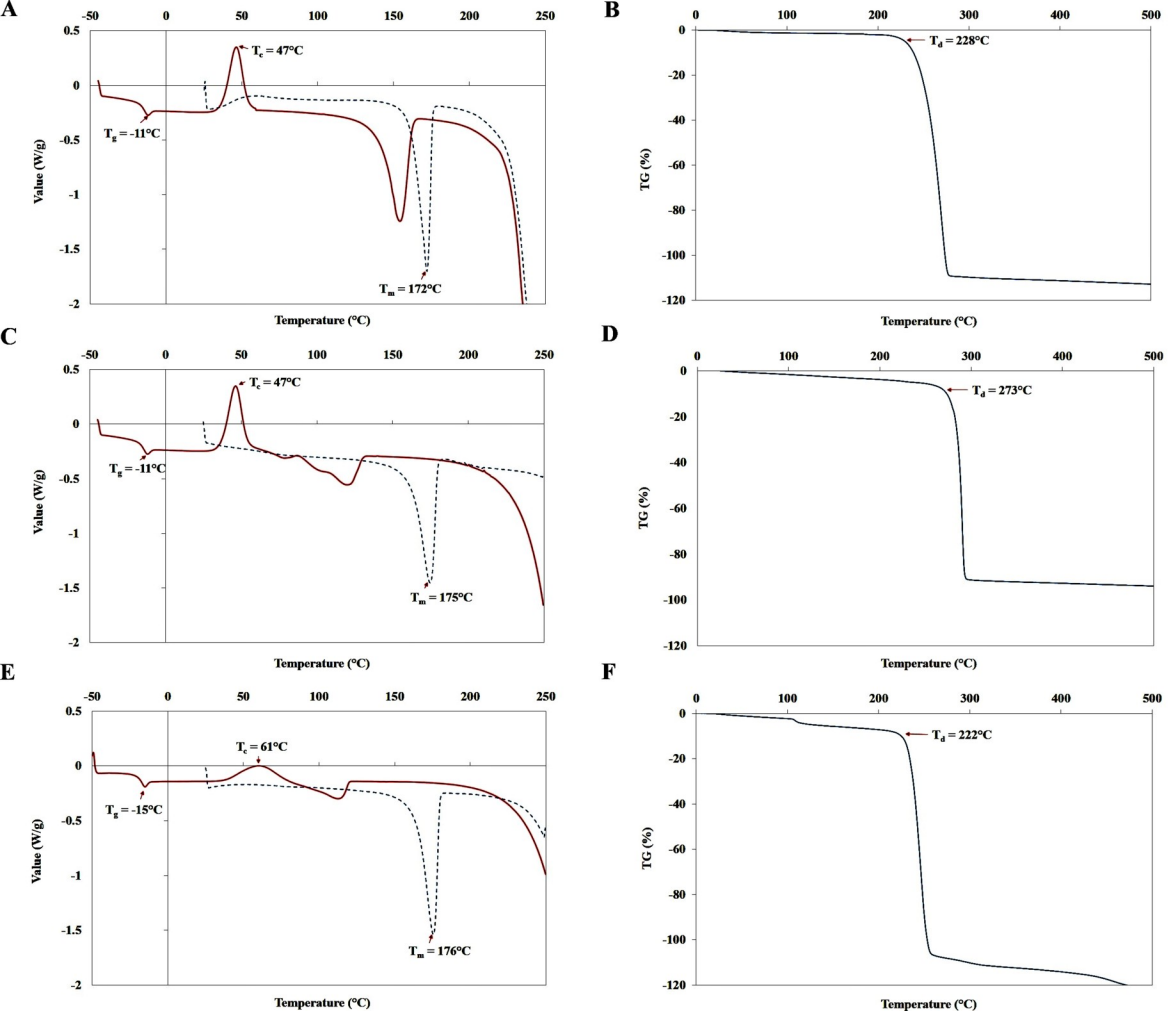
The melting temperature (T_m_), glass transition temperature (T_g_), crystallization temperature (T_c_) and degradation temperature (T_d_) of the PHAs obtained from EPPJ (A, B), glucose (C, D) and standard PHB (E, F) after DSC and thermal gravimetric analysis.

Thermal properties are of a vital consideration when selecting a polymer for packing. **Fig 6** represents a comparison of the TGA and DSC thermograms, for the PHB obtained using EPPJ, glucose and standard PHB. During the first heating scan from 25°C to 250°C, the PHB obtained from EPPJ and glucose exhibited melting temperature (T_m_) at 172°C and 175°C, respectively. This matched well with results obtained from the standard PHB with T_m_ = 176°C (**Fig 6E**). The second heating scan from -50°C to 250°C showed thermal crystallization temperature (T_c_) of 47°C, glass transition temperature (T_g_) of -11°C with 56.1% of polymer crystallinity in both EPPJ and glucose. After Thermal gravimetric analysis, the degradation temperature (T_d_) of the PHB obtained from EPPJ, glucose and standard PHB was 228, 273 and 222°C, respectively (**Fig 6B**, **6D** and **6F**). The DSC and TGA results in this study are similar to the values reported in previous publications [52, 78, 81]. High thermal stability of PHAs is an important parameter for polymerization process due to the polymer can withstand a high temperature.

Here, we introduce a new *Bacillus* strain, which was isolated and shown to produce high-levels of PHB using a low concentration of extracted pineapple peel juice (EPPJ), which is a cheap carbon source. This optimized medium can decrease the cost of PHB production and obtained good PHB properties similar to the commercial standard PHB.

## Conclusions

As an agricultural waste, pineapple peel is a cheap carbon source for PHB bioplastic production. In our experiment, 40.8 g/L reducing sugar and 5.6 g/L PHB were achieved using extracted pineapple peel juice (EPPJ) as a substrate in batch fermentation after being optimized by RSM. This relatively high PHB production indicates that EPPJ is a promising waste byproduct and can be used in the research and development of bioplastic.

## Supporting information

S1 Fig Fluorescent Nile red staining on mineral salt medium (MSM) agar plate containing 1% (w/v) glucose of isolated bacteria under UV light after incubated at 30°C for 3 days. (TIF)

S2 Fig Effect of different pH (A), inoculum size (B) and temperature (C) on PHAs production by Bacillus drentensis BP17 in optimized medium. The different letters show significant difference (p< 0.05).
(TIF)

S3 Fig Comparison of FTIR spectra of the polymers produced by *Bacillus drentensis* strain BP17 grown in TSB containing (----) EPPJ and NB containing (······) glucose with the (—) standard PHB. (TIF)

S4 Fig X-ray diffractogram of the polymers produced by *Bacillus drentensis* strain BP17 grown in TSB containing (----) EPPJ and NB containing (······) glucose with the (—) standard PHB. (TIF)

S1 Table 16S rRNA gene sequence similarity between selected PHAs-producing bacteria and their most closely related strains, intensity of fluorescence after staining with Nile red and PHAs content. (PDF)

S2 Table Differential characteristics of strain BP17 and phenotypically related species. Taxa: 1, strain BP17; 2, *Bacillus drentensis* LMG 21831^T^; 3, *B*. *cucumis* AP-6^T^; 4, *B*. *vireti* LG 21834^T^; 5, *B*. *novalis* LMG 21837^T^. Data were from this study unless indicated otherwise. Symbols: +, positive; W, weakly positive; -, negative; V, results vary between strains; ND, Not determined. All grassland isolates investigated in this study gave positive results for hydrolysis of aesculin and for acid production from *N*-acetyl-D-glucosamine, D-fructose, D-glucose and maltose. All strains gave negative results for arginine dihydrolase, lysine decarboxylase, ornithine decarboxylase, citrate utilization, hydrogen sulfide production, urease, tryptophan deaminase, indole production and acid production from D-arabitol, L-arabitol, dulcitol, erythritol, 2-keto-D-gluconate, methyl D-xyloside, L-sorbose, D-tagatose, xylitol and L-xylose.
(PDF)
