# Supplementary material for: Response surface method for polyhydroxybutyrate (PHB) bioplastic accumulation in Bacillus drentensis BP17 using pineapple peel
Source: PLoS One. 2020 Mar 19;15(3):e0230443. doi: 10.1371/journal.pone.0230443 (PMC7082031; doi:10.1371/journal.pone.0230443)
Supplement: S1 Table — (PDF) [file pone.0230443.s005.pdf]

Isolates	The most closely related strain	Length (bp)	Identity (%)	Intensity of fluorescence	Number of PHAs granules	PHAs content (%) of CDW
TM3-15	*Shinella fusca* DC-196^{T}	1,393	99.56	+++	2	5.6
TM12-4	*Shinella zoogloeoides* ATCC 19623^{T}	1,372	99.12	+++	2-3	18.9
AG5-2	*Shinella fusca* DC-196^{T}	1,378	99.63	++	3-4	18.7
AG5-3	*Mycoplana dimorpha* IAM 13154^{T}	1,367	99.71	+++	2-3	12.9
AG12-1	*Shinella zoogloeoides* ATCC 19623^{T}	1,358	99.71	+++	2	13.5
BP17	*Bacillus drentensis* LMG 21831^{T}	1,417	99.21	+++++	4-5	19.9
