# Supplementary material for: Response surface method for polyhydroxybutyrate (PHB) bioplastic accumulation in Bacillus drentensis BP17 using pineapple peel
Source: PLoS One. 2020 Mar 19;15(3):e0230443. doi: 10.1371/journal.pone.0230443 (PMC7082031; doi:10.1371/journal.pone.0230443)
Supplement: S2 Table — Taxa: 1, strain BP17; 2, Bacillus drentensis LMG 21831T; 3, B. cucumis AP-6T; 4, B. vireti LG 21834T; 5, B. novalis LMG 21837T. Data were from this study unless indicated otherwise. Symbols: +, positive; W, weakly positive; -, negative; V, results vary between strains; ND, Not determined. All grassland isolates investigated in this study gave positive results for hydrolysis of aesculin and for acid production from N-acetyl-D-glucosamine, D-fructose, D-glucose and maltose. All strains gave negative results for arginine dihydrolase, lysine decarboxylase, ornithine decarboxylase, citrate utilization, hydrogen sulfide production, urease, tryptophan deaminase, indole production and acid production from D-arabitol, L-arabitol, dulcitol, erythritol, 2-keto-D-gluconate, methyl D-xyloside, L-sorbose, D-tagatose, xylitol and L-xylose. (PDF) [file pone.0230443.s006.pdf]

Characteristics	1	2	3	4	5
Gram stain	+	+/v	+	-	+
Cell ends	Slightly tapered	Tapered	Rounded	Rounded/Slightly tapered	Rounded
Spore shape	Ellipsoidal/Circular	Ellipsoidal/Circular	Circular	Ellipsoidal	Ellipsoidal
Spore position	Paracentral (Subterminal)	Paracentral (Subterminal)	Subterminal	Central/Paracentral/(subterminal)	Subterminal(Paracentral)
Sporangia swollen	+	+	w	w	w
Growth at 50 ºC	-	+	-	-	+
Anaerobic growth	+	+	+	+	+
Hydrolysis of gelatin	-	-	-	+	v
Storage inclusions	+	+	+	-	-
ONPG	+	+	+	v	-
Urease	-	-	-	-	-
Nitrate reduction to N_2	+	v	-	+	+
Acid production from:					
L-arabinose	+	-	-	-	-
Amygdalin	+	v	-	-	v
D-cellobiose	+	-	-	-	v
Glycerol	-	-	-	-	v
Glycogen	-	-	-	+	-
Lactose	+	+	-	-	-
D-mannitol	+	-	-	+	v
D-melibiose	+	+	-	-	-
Methyl-α-D-glucoside	-	v	-	w	-
Raffinose	+	v	-	-	-
Ribose	-	v	-	w	v
Salicin	-	+	-	-	-
Starch	+	v	-	+	-
Sucrose	+	v	-	+	-
